# The Royal Sanitary Institute at Belfast

**Published:** 1911-07-29

**Authors:** 


### THE ROYAL SANITARY INSTITUTE AT BELFAST.

The Royal Sanitary Institute has been holding its Annual Conference at Belfast this year, at which several interesting papers were productive of equally interesting discussions. Dr. Parkes dealt with "The Prevention of Consumption as a National Task," and favoured the scheme laid down in the Insurance Bill. He laid stress on the importance of promoting co-operation between the Health Committees and local sanitary authorities, and the advisability of empowering the Treasury to advance money to local authorities at a very low rate of interest to enable them to improve housing and hygienic conditions in their districts. Sir T. Lauder Brunton dealt with the milk problem, and was inclined to press for a Pure Milk Bill and for better endowment of research. The other speakers referred to the immense advantage of an educative propaganda on behalf of hygiene and sanitation. The members have made several excursions and visited various institutions and places of interest in the neighbourhood.

Mr. Phillip Boobyer read a very interesting paper on "Municipal Hospitals," in which he pointed out that the Insurance Bill would enormously increase the demand for hospital accommodation and urged that provision should be made for municipalities to erect open-air hospital pavilions. Such provision is, however, already in existence, since the Public Health Act gives every local authority power to erect hospitals or to subsidise voluntary hospitals for special purposes in its district. Lord Aberdeen attended the Conference on the Hygiene of Childhood, at which papers were read on school hygiene, infant mortality, and the sanitation of schools. Not the least interesting feature of the conference has been the admirable lay lecture on the prevention of consumption, delivered by Dr. Parkes, referred to above.

